# Evacuation Difficulties : The Triumph of the Motor Ambulance

**Published:** 1915-01-02

**Authors:** 


### THE WORK OF THE R.A.M.C. IN WAR.

### Evacuation Difficulties: The Triumph of the Motor Ambulance.

BY AN OFFICER OF THE R.A.M.C. AT THE FRONT.

THE keynote of the successful working of the system for the disposal and treatment of the wounded in war-time is "Evacuate, evacuate, evacuate." Thus the regimental medical officer, having established his "aid post" a few hundred yards in rear of the firing-line, at present synonymous with the advanced trenches, will soon find the accommodation at his disposal insufficient for the number of sick and wounded to be dealt with, unless his aid post is regularly and frequently "evacuated," or, in other words, cleared of the patients. It is for the field ambulance to manage this, since the regimental medical officer has no waggons of his own for the purpose; and this is why it is so vitally important for him to notify the field ambulance of his whereabouts, describing exactly in writing the position of the house, barn, farm, or cottage in which he has established his aid post. (In summer-time a covered place is not essential; the lee side of a haystack, or some similar shelter, is as good, and has the advantage of being less liable to draw shell-fire, and safer if it does draw it.)

In their turn, the field ambulances must endeavour with all speed to "evacuate" the patients who have been collected from the various aid posts. At the dressing station the cases are kept just long enough for redressing (when necessary), for operations of urgency, and for such attentions as hot food, washing, replacement of fouled garments by hospital clothing, and so forth. As soon as these attentions have been bestowed on the patient he is ready for "evacuation." The reason for this urgency is that the field ambulance has to conform to the movements of the troops. This cannot be done if a large number of patients is under care, or rather, it can only be done by leaving the patients to take their chance—a contingency which might arise and is provided for in the official text-books of instruction of the Royal Army Medical Corps. Thus, in order to preserve their mobility, the field ambulances must be perpetually "evacuated" of patients. We shall return presently to the details of this problem, one of the most vital and crucial of the whole service of aid to the wounded. To pass on for the moment: the wounded from the field ambulances are taken to clearing hospitals, which are established usually at railhead, or as near it as possible. Here a certain degree of assortment of the patients can be attempted, but in the main the "evacuate" rule holds good, and the clearing hospitals are emptied as regularly and quickly as possible, as a rule, by ambulance trains, which in turn "evacuate" themselves into base hospitals or general hospitals on the way to the base. From these the wounded are sooner or later "evacuated" once more to England, or, if only slightly ill, into convalescent hospitals, whence they return to the Front.

To return to the matter of removal of the wounded from the field ambulances to the clearing hospitals. This is the link in the chain which at the outbreak of war and for some weeks afterwards was the weakest, but is now one of the strongest. The great difficulty in August and September was due to the fact that clearing hospitals, not being mobile units, are not provided with transport. This involves two corollaries: one, that clearing hospitals cannot get nearer to the "Front" than railhead; the other, that they cannot send out to the dressing stations of the field ambulances to evacuate thence the patients, but must be mere passive agents ready to receive anyone brought to them, but not able to do any active collecting. Now the first of these corollaries means that during an advance by a victorious army —e.g. round about the Marne—the dressing stations formed by the field ambulances on the battlefields are separated from the nearest clearing hospital by anything up to ten or twenty miles. And the second means that the field ambulances must by hook or by crook manage to bridge the gap and get their cases to the clearing hospital somehow.

Now during the early weeks of the war, as has been said, this gap presented great difficulties in securing successful and continuous evacuation. Thus after the battle of the Marne there was great difficulty in the evacuation of patients from the field ambulances, which were ordered to push on after the advancing army. In one ambulance, which had picked up well over a hundred wounded, the solution adopted was as follows: Three medical officers—that is, one-third of the medical officers of the ambulance—and a dozen orderlies were left behind in charge of the wounded, who were distributed among a small village school and four adjacent barns. A quantity of medical stores, food, cooking-pots, stretchers, and so forth had also to be left; and this involved leaving a forage-cart and a water-cart for their transport. The necessary drivers and servants totalled eight, so that there were in all three officers and twenty men detached, left in a remote French hamlet with a hundred-odd wounded on their hands and no clearing hospital within fifteen miles. What was to be done? The food supply for the wounded was not more than enough for two days, and several of them were in urgent need of better accommodation than could possibly be given them. Fortunately, a mile or so away ran a high-road, leading in the direction taken by the troops one way, and back to railhead the other. So a sentry was posted on this road with orders to stop the first convoy of supply lorries and to present a note to the officer in command of them. As it happened, a convoy presently came along full of supplies for the troops. The officer in charge, duly stopped by the sentry, promised to call in on his return trip with the empty lorries, and this promise he kept some fifteen or sixteen hours later. Straw was spread on the lorries and the wounded were loaded upon them: not an ideal method of transport by any manner of means, for lorries driven at considerable speed over jolty country roads are not exactly ideal ambulance waggons. One of the medical officers was put on also, in medical charge, and the other two had then to devote themselves to the task of packing their stores and of trekking on after the main body, with the drivers and R.A.M.C. *personnel*. This involved four or five days of hard travelling, rendered especially difficult by the entire lack of any reliable information as to the course taken by the particular division to which the field ambulance in question belonged.

This actual case illustrates one of the ways in which the evacuation problem used to be surmounted. There were other ways of achieving the same end, such as by detaching from the field ambulance the number of ambulance waggons necessary to carry all the wounded and sending them back by road to the clearing hospital. This course was only practicable when the number of patients to be evacuated was small, and it involved the same disadvantages as the system already described, and on a larger scale. In this method not only does the field ambulance lose for some days the services of a high proportion of its medical officers and of a certain number of trained rank and file, but it also loses during that time the use of some or all of its ambulance waggons, and is thereby deprived to a corresponding extent of its proper function of evacuating the aid posts, should another action supervene before the missing waggons rejoin. In the writer's own actual experience this plan was adopted only once, at a moment when three ambulance waggons sufficed to evacuate the whole of the patients admitted to the field ambulance, and it was vitally important that the main body of the latter should move off without the slightest delay.

Yet another method of evacuation is to leave behind in charge of a depôt of wounded an officer and a few men with orders to commandeer from amongst the neighbouring farms and villages the carts and horses necessary to accommodate the wounded; and then to convoy the whole lot down to the clearing hospital. It is advisable when pursuing this course to take the owners of the requisitioned transport to act as drivers; and they can then be allowed to return to their homes with their carts and horses as soon as the wounded are shifted. Another way which might conceivably be necessary is simply to leave the wounded in the dressing station with a couple of orderlies in attendance, and to notify the General (through the Assistant Director of Medical Services) of their location, leaving him to make such arrangements as he could for their removal. During the retreat from Mons towards Paris yet another variety of evacuation was sometimes practised. Owing to the continual retirement all sorts of lines of communication units, such as general hospitals, clearing hospitals, and others, found themselves a good deal nearer the firing-line than they had any business to be in the ordinary way. It thus happened a good many times that field ambulances retiring with their waggons full of wounded, and seeking some way of evacuating them, would arrive at some place where a clearing hospital with an ambulance train was installed. Thus the ambulances were several times enabled to evacuate straight into ambulance trains, which would steam away in triumph just before the arrival of the pursuing Germans.

### The Motor-Ambulance.

It will have been noticed that so far the past tense has been exclusively used in speaking of the various devices used to secure prompt evacuation, for these makeshift methods have all been superseded by the advent of the motor-ambulance. This invaluable conveyance plies backwards and forwards between the dressing station and the clearing hospital, achieving with celerity, certainty, and comfort for the wounded results which were quite impossible in the earlier stages of the war. The British Expeditionary Force, in spite of the repeated warning advice in recent years of experienced medical organisers, possessed no motor-ambulances when it first left our shores, and it was left for private benevolence (at first) to provide these absolutely essential ingredients of an efficient medical aid service. The St. John Ambulance Association and the British Red Cross Society rose nobly to the occasion. When our divisions were moved from the Aisne to the Flanders battlefield the motor-ambulance service was in a thoroughly organised state of high efficiency, and the War Office had already begun to send cars out to share in the work as part of the *matériel* of the R.A.M.C. By this time the War Office cars outnumber those of the voluntary agencies, and are doing a most admirable work.

The motor-ambulance, in other words, has solved the problems of evacuation completely. It is not, as a rule, permitted to go up to the regimental aid post, nor even to the advanced dressing station of the field ambulance. Broadly speaking, the Assistant Directors of Medical Services (under whose direct orders the motor-ambulances work) are very chary of allowing the cars to go to any place at which there is more than an infinitesimal risk of their receiving shell fire. This care for the motor-ambulances is all very right and proper, since a motor engine is very easily disabled, and the car is practically immobile if such an accident happens. But now that the supply of cars is so abundant that only a part of the total are required for work at any one time, this cautious policy is no longer necessary and is, in fact, being pushed somewhat to excess. The wounded have to be removed somehow or other, and motor-transport, being far speedier than horse-waggons or bearers, exposes the wounded to far smaller risks from hostile fire *en route* than does any other kind. For the same reason the *personnel* of the R.A.M.C. are far less exposed to injury by the use of motor-transport, since a much smaller number of men can do the same work in a far shorter time.

